# Endometriosis: surgical management for fertility enhancement

**DOI:** 10.3389/fendo.2026.1861188

**Published:** 2026-07-22

**Authors:** Danielle A. Aase, Zaraq Khan

**Affiliations:** Department of Obstetrics and Gynecology, Mayo Clinic, Rochester, MN, United States

**Keywords:** deep endomertriosis, endometriosis, fertility, infertility, surgery

## Abstract

Endometriosis is a common, chronic inflammatory condition characterized by the presence of endometrium-like tissue outside of the uterus that can impact fertility through multiple mechanisms. In this review, the diagnosis, classification, staging, and surgical management of endometriosis for fertility enhancement is discussed. Surgery for patients seeking to optimize fertility or for whom infertility is the only symptom is complex. In patients with superficial endometriosis, laparoscopy may enhance fertility for unassisted pregnancy, but current guidelines recommend against routine surgery prior to ART. In patients with deep endometriosis, surgical intervention depends on multiple factors, including patient age, ovarian reserve, fertility goals, and the presence of other infertility factors. While there may be potential improvements in fertility-related outcomes, this must be weighed against potential impacts to ovarian reserve. Surgical techniques that minimize damage to the ovaries, fallopian tubes, and uterus are essential when fertility is the goal. While there is increasing interest in regenerative therapies for treatment of endometriosis (e.g., stem-cell based therapies, platelet-rich plasma, or extracellular vesicles), these remain investigational and are not yet utilized clinically for fertility enhancement in patients with endometriosis.

## Introduction

Endometriosis is a common, chronic inflammatory condition characterized by the presence of endometrium-like tissue outside of the uterus (1, 2). While it is estimated that approximately 10% of reproductive-aged individuals with a uterus are affected, many factors may lead to an underestimate of the true prevalence rates across different populations (1, 3). These include demographic, economic, or social barriers, such as stigma or lack of awareness (3). Although patients can be asymptomatic, most patients present with pelvic pain, adnexal mass, or infertility (2). Risk factors for endometriosis include low body mass index (BMI), alcohol use, smoking, mullerian anomalies, first-degree relative with endometriosis, and prolonged endogenous estrogen exposure as seen in early menarche, nulliparity, or short menstrual cycle interval (1, 2, 4). Greater parity and longer length of total breastfeeding have been shown to be protective factors (5). In addition to increased risk of ovarian cancer and pregnancy complications, endometriosis has been increasingly recognized as a systemic condition with impacts on non-reproductive organs, hormone metabolism, cardiovascular disease, and autoimmune disorders (1). Genetic factors have also been identified; one study of twins estimated the heritability of endometriosis to be around 50% (6, 7). The observed inheritance patterns suggest polygenic or multifactorial genetic factors, rather than a single gene mutation (8).

While a causal relationship between infertility and endometriosis has not been clearly established, the association between infertility and endometriosis is well known. Previous studies have suggested that 30-50% of patients with endometriosis have infertility, and 25-50% of patients with infertility have endometriosis (2). Natural fecundity may decrease to 2-10% in couples with endometriosis (1). There are several mechanisms that have been proposed to explain endometriosis-associated infertility.

### Distorted pelvic anatomy

Patients with advanced stage endometriosis can have pelvic adhesions that block fallopian tubes, impair oocyte release from the ovary, or impede oocyte capture or transport (2, 9).

### Altered peritoneal function

Patients with endometriosis have been shown to have higher peritoneal concentrations of inflammatory cytokines such as IL-1, IL-6, and TNF-α and angiogenic cytokines such as VEGF (2, 9). This chronic inflammation may reduce ovarian response and impair sperm motility (9). In addition, these cytokines may impact fertilization, embryo development, and implantation (2, 9).

### Endocrine and ovulatory abnormalities

Patients with endometriosis may have a longer follicular phase, lower serum estradiol levels, and lower LH-dependent progesterone secretion during the luteal phase (2, 9). This may impair folliculogenesis and lead to reduced oocyte quality (9). Patients with endometriosis may also have a higher prevalence of luteinized unruptured follicle syndrome, a condition in which a follicle undergoes luteinization but fails to rupture and release an oocyte (2, 9). Finally, patients with endometriosis may have luteal phase deficiency, in which the corpus luteum does not produce an adequate amount of progesterone resulting in a shortened luteal phase (10).

### Impaired implantation

There have been some studies that suggest that patients with endometriosis may have disorders of endometrial function and impaired embryo implantation (2, 9). One study demonstrated decreased endometrial expression of αvβ3 integrin (a cell adhesion molecule) during the time of implantation in patients with endometriosis (11). Other proposed mechanisms include altered expression of other implantation factors, such as HOXA10, or decreased endometrial response to progesterone due to progesterone receptor resistance (9). Finally, luteal phase deficiency may impair implantation due to embryo-endometrial asynchrony (10).

### Gamete and embryo quality

Patients with endometriosis may have reduced oocyte and embryo quality. Patients with endometriomas (endometriosis ovarian cysts) may experience accelerated follicle depletion leading to diminished ovarian reserve (9). One study showed that embryos from patients with endometriosis developed more slowly and had a higher rate of arrested development compared to those with tubal factor infertility (12). Another study examining donor oocyte cycles showed that patients with endometriosis who received oocytes from disease-free patients had normal implantation and pregnancy rates. However, when patients without endometriosis received oocytes from donors with endometriosis, implantation and pregnancy rates were decreased (13).

### Abnormal uterotubal transport

Patients with endometriosis may have reduced uterotubal transport function. In one study, hysterosalpingoscintigraphy (HSSG) was utilized to evaluate uterotubal transport function in patients with endometriosis and patent fallopian tubes compared to controls with male factor infertility. There was a higher rate of abnormal tubal transport in 64% of patients with endometriosis compared to 32% of controls (14).

### Dyspareunia

Dyspareunia, a common symptom of endometriosis, can have a significant impact on patients’ sexual function and quality of life. Prior studies have shown that patients with endometriosis and dyspareunia have reduced quality of life across multiple domains, including physical function, general health, mental health, and social functioning (15). Dyspareunia can be classified as superficial (discomfort or pain at the vaginal introitus or with vaginal entry) or deep (discomfort or pain with deeper penetration). Severe dyspareunia may significantly impact sexual behavior and reduced frequency of intercourse may lead to reduced chance of pregnancy (16).

The treatment of infertility in patients with endometriosis can be challenging: medical suppression of endometriosis with hormonal medications is a cornerstone for pain management, but these cannot be used in patients actively trying to conceive. As such, options for fertility enhancement in patients with endometriosis include expectant management, assisted reproductive technology (ART), surgery, or a combination of surgery and expectant management or surgery and ART (2, 9). Decision-making between options is nuanced and depends on multiple clinical factors. Therefore, the primary objective of this review is to provide a broad overview of surgical treatment of endometriosis for fertility enhancement in patients with both superficial and deep endometriosis.

## Diagnosis and classification of endometriosis

**Figure 1:**
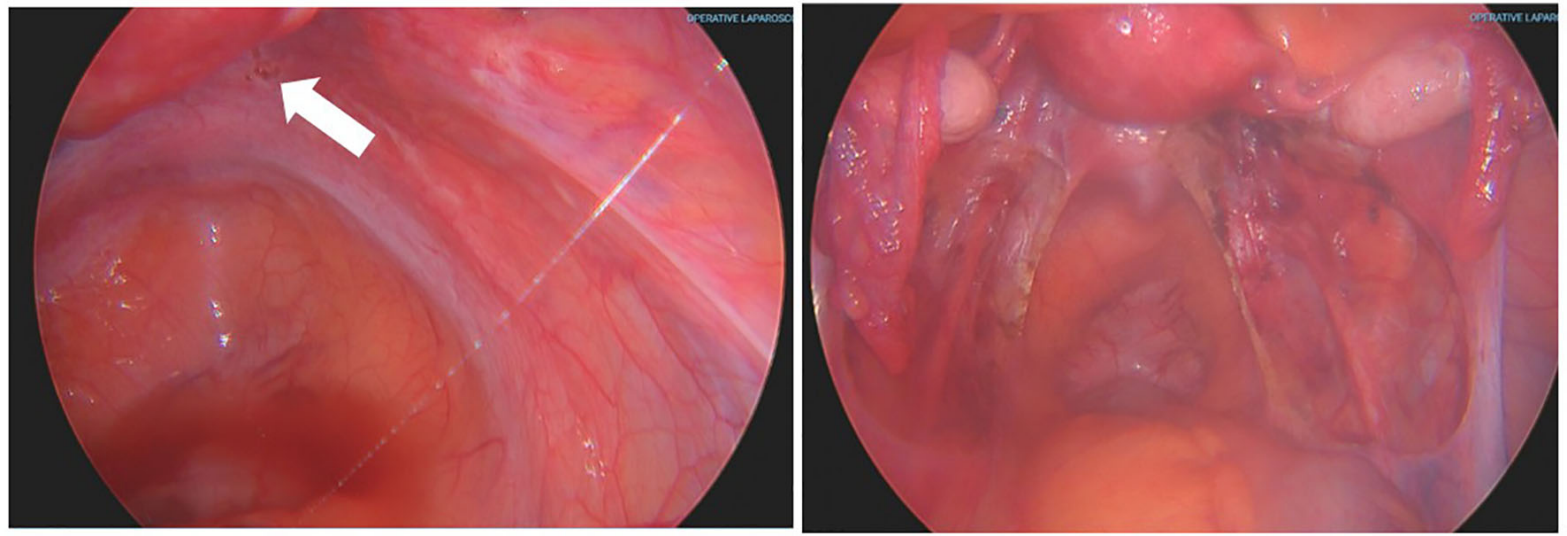
Left: View of right pelvic sidewall and posterior cul de sac with stage I endometriosis involving the right uterosacral ligament. Right: View of pelvis after bilateral ureterolysis and complete disease resection.

**Figure 2:**
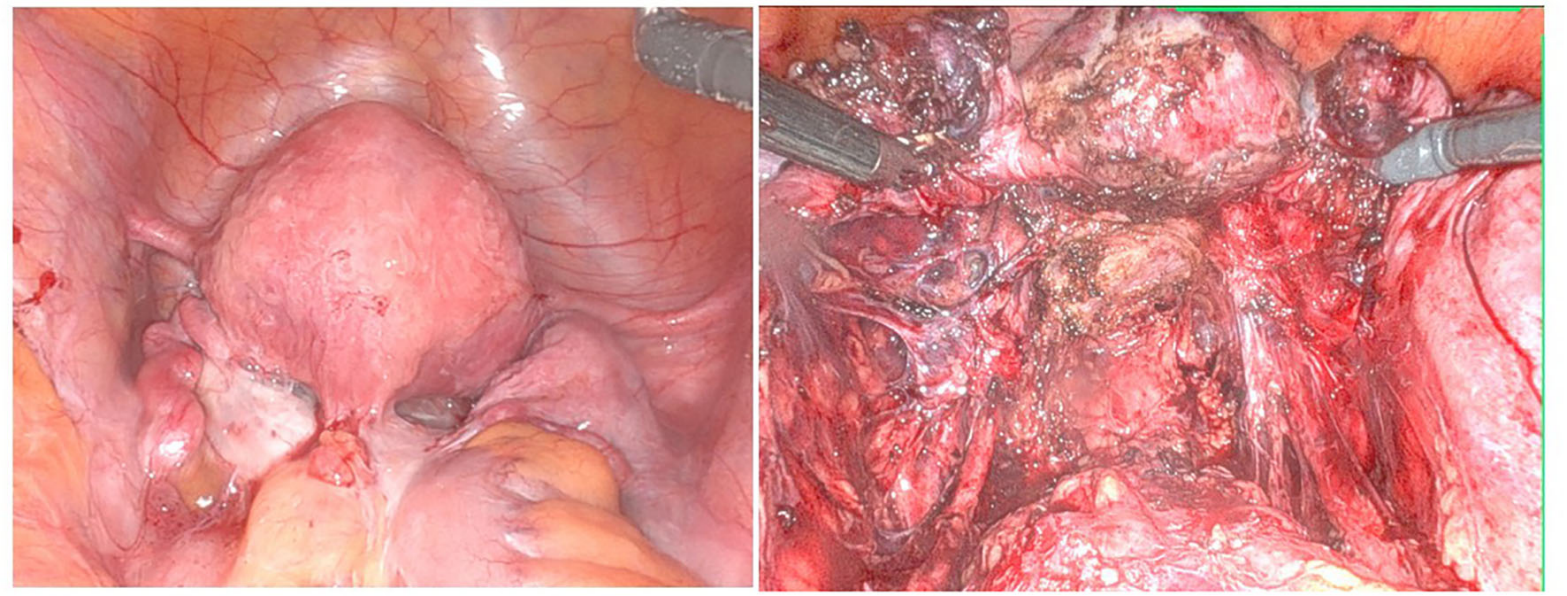
Left: Stage IV endometriosis and DE with bilateral endometriomas, complete obliteration of the cul-de-sac, bilateral hydrosalpinx, and superficial rectal disease. Right: View of pelvis after complete disease resection, which included bilateral salpingectomy and ovarian cystectomies, ureterolysis, low anterior resection of rectum with primary end to end anastomosis, ureterolysis, and excision of pelvic peritoneum.

In current clinical practice, visual or histopathologic confirmation of endometriosis is the current gold standard for diagnosis (4). However, advances in imaging dedicated to detection of early and advanced stage endometriosis allow it to be used clinically for diagnosis, along with patient history, symptoms, and physical examination (4, 17, 18). Therefore, imaging has the potential to replace histopathology as means of standardized diagnosis in the near future. Histopathologic diagnosis is often achieved by a surgical procedure such as laparoscopy (2). There are four main subtypes of endometriosis: superficial, deep endometriosis (DE), ovarian, and extrapelvic endometriosis. Superficial endometriosis lesions occur on peritoneal surfaces within the pelvis (**Figure 1**). Deep endometriosis lesions penetrate pelvic peritoneal surfaces or extend into the muscularis propria of pelvic organs (**Figure 2**). Endometriomas are ovarian cysts lined by endometrial glands. Finally, extrapelvic endometriosis refers to endometriosis outside of the pelvis (4).

### Diagnostic tools

Assessment of disease extent with imaging is important not only for guiding treatment options but also in assisting with surgical planning. Following history and physical examination, all patients require imaging with either pelvic ultrasonography or magnetic resonance imaging (MRI). While superficial peritoneal disease is harder to detect with imaging, endometriomas and DE can be detected much more readily.

**Figure 3:**
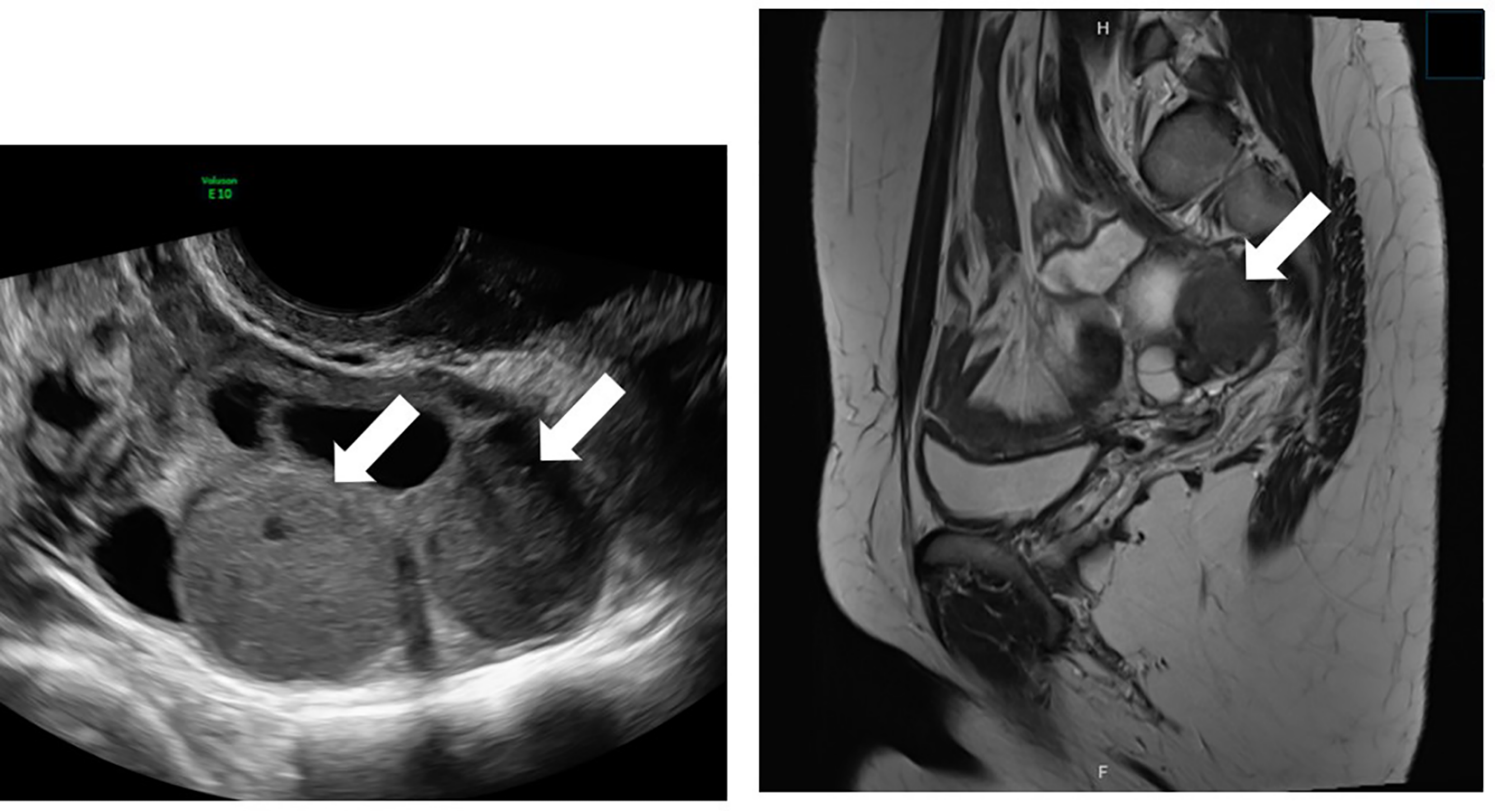
Left: Transvaginal ultrasound image of right ovary with two endometriomas. Right: Sagittal view of T2-weighted MR image of the same ovary with T2-hypointense lesion representing endometrioma.

Pelvic ultrasonography is the preferred initial imaging modality for patients with suspected endometriosis due to its availability, lower cost, and ability to identify other pelvic pathology (1). However, dedicated endometriosis protocols are needed. In 2016, the International Deep Endometriosis Analysis (IDEA) group published consensus guidelines on the sonographic evaluation and terminology of endometriosis. They propose four sonographic steps in the evaluation of patients with known or suspected endometriosis, including 1) routine evaluation of the uterus and adnexa 2) evaluation of transvaginal sonographic “soft markers” (e.g., ovarian mobility) 3) assessment of the pouch of Douglas with the uterine sliding sign and 4) assessment of endometriosis nodules in the anterior and posterior compartments (19). Standard pelvic ultrasonography has the highest sensitivity and specificity for diagnosis of endometriomas (93% and 96%, respectively), which classically appear as a unilocular cyst with homogenous low-level echogenicity (**Figure 3**) (20). When evaluating for deep endometriosis, sensitivity of standard pelvis ultrasound is moderate (79%) (20). Sensitivity may be improved with augmented pelvic ultrasound; a prospective observational study found that pelvic ultrasound augmented with the IDEA consensus guidelines had a sensitivity of 88% for the detection of deep endometriosis (21).

When pelvic ultrasonography is ambiguous and additional information is needed for surgical planning, MRI can be obtained to evaluate disease distribution. It is also helpful for assessing extrapelvic disease. Similar to pelvic ultrasonography, dedicated imaging protocols for endometriosis are key. Disease detection can be improved through techniques such as vaginal distension with ultrasound gel, partial bladder distension, and the use of antiperistaltic agents such as glucagon or hyoscine butylbromide (22). DE appears as T2-hypointense and/or T1-hypertense areas on MR images in any of the following areas: the torus uterinum, uterosacral ligaments, vagina, rectovaginal septum, rectosigmoid, pouch of Douglas, parametrium, bladder, and round ligaments (23). MRI is more useful for evaluation of suspected DE, with a sensitivity and specificity of 94% and 77%, respectively (20).

### Classification systems

Given the heterogeneity of endometriosis – ranging from 1 mm superficial peritoneal implants to large endometriomas and posterior cul de sac obliteration – several staging systems have been developed to facilitate communication about disease extent and to allow comparison of research outcomes. The most common staging system is the revised American Society for Reproductive Medicine classification system for endometriosis (rASRM). This system classifies endometriosis as minimal, mild, moderate, or severe (stage I-IV, respectively) based on the size and location of disease lesions (24). However, it does not address surgical complexity and has limited correlation with pain symptoms (25). Therefore, other staging systems have been developed. The Endometriosis Fertility Index was designed to predict unassisted pregnancy rates after surgical staging in patients with infertility and endometriosis (26). The Enzian classification system was designed to facilitate classification of DE without a points-based score (27). Finally, the AAGL 2021 Endometriosis classification system was developed as an anatomy-based and user-friendly scoring system that correlates with surgical complexity (25). Each staging system has benefits and limitations and may be applied in different clinical scenarios depending on the desired treatment outcome.

## Surgery for fertility enhancement in endometriosis

Surgery for endometriosis-related pain can be effective for symptom management. However, the decision-making for surgery for fertility enhancement/optimization can vary based on the patient’s age, symptoms, clinical history, and fertility goals and is more complex (1). Patients with clinical signs or symptoms of endometriosis such as cyclic or chronic pelvic pain, painful menstrual cycles, or pain with intercourse as well as infertility would most likely benefit from a diagnostic laparoscopy to diagnose and treat disease, if present (9). In the absence of any clinical signs or symptoms of endometriosis, diagnostic laparoscopy is not recommended for patients with infertility simply to rule out or confirm disease (2). Management differs based on the presence of superficial (stage I/II, minimal/mild) disease versus deep (stage III/IV, moderate/severe) disease, including endometrioma and disease of non-reproductive organs such as the bowel, urinary tract, and/or extrapelvic locations.

### Superficial endometriosis

Superficial endometriosis can be treated with electrocautery ablation or excision with removal of the affected peritoneum (1). Though no significant outcome differences are noted in the literature, excision of disease is preferred over ablation of lesions. While excision of disease may improve pain symptoms, the effect on fertility is nuanced. For stage I/II disease associated with infertility, laparoscopic surgery for treatment of endometriosis may enhance fertility for unassisted pregnancy (2, 17, 28–30). The most recent ESHRE (European Society of Human Reproduction and Embryology) guidelines make a weak recommendation for operative laparoscopy as a treatment option for endometriosis-associated infertility in stage I/II endometriosis as it may improve the rate of ongoing, unassisted pregnancy (17). This recommendation is largely based on a systematic review and meta-analysis of three randomized controlled trials (RCTs) that concluded that laparoscopic treatment of stage I/II endometriosis may improve viable intrauterine pregnancy rates compared to diagnostic laparoscopy alone (OR 1.89; 95% CI 1.25-2.86) based on moderate quality evidence (30). Similar conclusions were drawn from another meta-analysis of four trials on treatment of stage I/II endometriosis, which showed an increase in pregnancy rates following treatment (RR 1.44; 95% CI 1.24-1.68). When analysis was restricted to RCTs only (two trials) the conclusion remained similar (OR 1.44; 95% CI 1.06-1.95) (31). When the results of these two RCTs are combined, the number needed to treat is 12. In other words, for every 12 patients with stage I/II endometriosis, there will be one additional successful pregnancy with treatment of visible lesions compared to no treatment. However, it is important to note that this improvement would only apply to patients with endometriosis. If the true estimated incidence of endometriosis in otherwise asymptomatic patients with unexplained infertility is applied, then the number needed to treat for one additional pregnancy is actually 40 (2).

This high number needed to treat favors fertility treatments over surgical management for patients with stage I/II endometriosis and infertility (1). One retrospective study compared assisted reproductive technology (ART) outcomes for patients following laparoscopic treatment of stage I/II endometriosis versus diagnostic laparoscopy only. Patients who had removal of all visible endometriosis prior to ART had significantly higher implantation, pregnancy, and live birth rates (OR 1.47; 95% CI 1.01-2.13) compared to controls (32). While this result suggests there may be a benefit of surgery for stage I/II endometriosis prior to ART, the most recent ESHRE guidelines recommend against routine surgery prior to ART to improve live birth rates until further studies are completed (17). However, surgery remains a viable option for fertility seeking patients with superficial disease as well as significant symptoms related to endometriosis.

### Deep endometriosis

Surgical intervention for stage III/IV (moderate/severe) endometriosis for fertility enhancement is complex. This includes deep disease of reproductive organs as well as of non-reproductive organs such as the bowel, urinary tract, or extrapelvic disease such as disease in the thoracic cavity. Some observational studies have suggested a potential beneficial impact of complete disease resection on reproductive outcomes, both on chances of unassisted pregnancy and for ART outcomes (33). A systematic review and meta-analysis of five studies comparing reproductive outcomes in patients with infertility and DE who received *in vitro* fertilization (IVF) with or without a previous surgery for DE showed a statistically significant benefit of surgery prior to IVF both with (OR 2.43; 95% CI 1.13-5.22) and without (OR 1.55; 95% CI 0.61–3.95) bowel involvement (34). However, a recent systematic review and meta-analysis compared surgery followed by IVF versus direct IVF on reproductive outcomes in patients with infertility and deep endometriosis and endometriomas. Surgery followed by IVF did not improve the live birth rate or clinical pregnancy rate in patients with deep endometriosis or endometriomas compared to a direct IVF approach. The authors concluded that IVF should be prioritized for these patients, reserving surgery for symptom management or in specific clinical situations to optimize reproductive outcomes and patient safety (35). There are no published RCTs that evaluate whether clinical pregnancy or live birth rates improve after treatment of stage III/IV disease. However, this gap in the literature is currently being addressed in the Surgery versus IVF for the treatment of infertility associated to ovarian and deep endometriosis (SVIDOE) multicenter, randomized controlled trial (36). Preliminary findings have suggested a benefit to IVF over surgery, with both intention-to-treat and per-protocol analyses showing a significantly higher live birth rate (p=0.009 and p=0.005, respectively) (37). While this is emerging evidence and a full report is still forthcoming, these findings may support a recommendation for IVF first prior to surgery.

In summary, patients with infertility and stage III/IV disease are likely to require ART over a trial of unassisted pregnancy, either as a primary treatment for infertility or immediately after surgery. Patients may be counseled towards surgery if they have significant pain, presence of hydro/hematosalpinx, large endometriomas, constriction of the ureter or bowel, or thoracic disease (i.e., recurrent pneumo/hemothorax). Patients with advanced reproductive age, low ovarian reserve, tubal or male factor, or a combination of these could consider immediate IVF before surgical intervention.

### Endometrioma and endometriosis of the fallopian tubes

Endometriomas can be found in approximately 20-40% of patients with endometriosis and are associated with more advanced disease. They are thought to develop through the invagination of endometriosis tissue on the ovarian serosa (38). Treatment of ovarian endometrioma in the setting of infertility is often not clear. The decision for surgery for endometriomas depends on the patient’s age, ovarian reserve, fertility goals (infertility history, ideal family size, desire/ability to do IVF), the size, location, and number of endometriomas (38). If patients wishing to conceive with no other infertility factors are incidentally noted to have endometrioma without concern for malignancy, they can attempt unassisted conception for up to 6 months before seeking specialist consultation (38). If surgery is considered, the potential impact on ovarian reserve must be weighed against potential benefits of improvement of symptoms facilitating unassisted conception or improved ovarian access during IVF.

Ovarian lesions can be addressed in one of three different ways: (1) incision and drainage (2) ablation or (3) laparoscopic cystectomy. Incision and drainage involves incision of the endometrioma to allow the cyst contents to drain, without removal of the cyst wall. Ablation of the endometrioma can be achieved through multiple methods, including ethanol sclerotherapy, monopolar, bipolar, plasma, or CO2 laser (39). Incision and drainage or ablation of endometrioma may impact ovarian reserve less than cystectomy, but recurrence rates are higher (40). Ethanol sclerotherapy may be beneficial to preserve ovarian reserve while treating endometrioma, but more data is needed before recommending it as a routine procedure for patients seeking to preserve fertility.

Cystectomy involves dissection of the cyst wall away from the ovarian stroma and excision of the cyst in its entirety. Cystectomy has been associated with reduced recurrence of symptoms such as dysmenorrhea, dyspareunia, and non-menstrual pelvic pain, reduced recurrence of the endometrioma, and a reduced requirement for further surgery. However, it has also been shown to reduce ovarian reserve to a larger extent than the other methods (40, 41).

Studies examining reproductive outcomes after cystectomy have variably defined large endometriomas as 3 or 4 cm or greater. One study found that laparoscopic cystectomy for ovarian endometriomas > 4 cm improved fertility compared to cyst drainage (42). A systematic review and meta-analysis found that laparoscopic management of ovarian endometrioma > 3 cm in patients with infertility was associated with a subsequent increased rate of unassisted pregnancy (OR 5.2; 95% CI 2.04-13.29) (40). However, while data from this systemic review and meta-analysis showed benefit for excision of endometriomas prior to unassisted pregnancy, the overall evidence does not support endometrioma surgery prior to IVF (39). Excision of endometriomas have been associated with a decrease in ovarian reserve, with those undergoing bilateral cystectomy showing the greatest decline (41). One retrospective, matched case-control study compared ART outcomes in patients who had undergone laparoscopic cystectomy for endometriomas > 3 cm to patients with endometriomas of a similar size who had not undergone surgery and found that fertility outcomes were similar (43). As such, current guidelines do not recommend cystectomy for asymptomatic patients with endometriomas prior to IVF (2, 17). However, it is prudent to weight risks and benefits of laparoscopic cystectomy prior to IVF, especially for larger endometriomas > 3–4 cm. Potential benefits include reduction in pain and symptoms, facilitation of oocyte retrieval, prevention of possible endometrioma rupture, and detection of occult malignancy (2). A systematic review published in 2021 proposed a treatment paradigm for endometrioma when fertility is desired. Unassisted conception was recommended for endometrioma < 3–4 cm and optional oocyte/embryo cryopreservation was recommended prior to cystectomy for endometrioma ≥ 3–4 cm (44). The recommendation for oocyte/embryo cryopreservation prior to cystectomy assumes normal ovary tissue is accessible for oocyte retrieval. In some cases, cystectomy may be needed first to facilitate oocyte retrieval.

Endometriosis may also affect the fallopian tubes, causing fibrotic fimbria, tubal blockage, and hemato/hydrosalpinx (1). If these are seen, the affected fallopian tube should be removed. Studies have shown that implantation is decreased by 50% in patients undergoing IVF with hydrosalpinx compared to without. Furthermore, salpingectomy for hydrosalpinx prior to IVF leads to improved implantation, pregnancy, and live birth rates (45). Rarely, a fimbrioplasty could be attempted if the patient has no means to complete IVF and the hydrosalpinx is < 4cm dilated. However, this is associated with a high risk of ectopic pregnancy up to 10% (1, 46).

### Bowel endometriosis

Bowel endometriosis affects approximately 10% of patients with DE and has been associated with pelvic pain and infertility. Management of infertility in patients with bowel endometriosis includes ART or primary surgical intervention. Decision making is nuanced and depends on the patient’s age, symptoms, co-existing infertility factors, and surgical history (33). Current guidelines do not recommend surgery before ART in patients with bowel endometriosis with a primary goal of improving fertility (17). While some studies show a benefit to surgery prior to ART, other studies show no difference in fertility outcomes in patients with untreated bowel endometriosis compared to patients without endometriosis undergoing IVF (33, 47). One common limiting factor in many of these studies is the lack of differentiation of patients who have co-existing adenomyosis, which has been associated with a detrimental impact on fertility. One study showed a lower pregnancy rate in patients with adenomyosis compared to those without following surgery for DE, including bowel endometriosis (48). Overall, evidence on surgery for bowel endometriosis for fertility enhancement – including before ART – is mixed and shared-decision making incorporating the patient’s clinical history and symptoms should be emphasized.

### Urinary tract endometriosis

Endometriosis may also affect the urinary tract. Most endometriosis of the urinary tract affects solely the peritoneum overlying the bladder or ureter but also may present with direct bladder, ureter, or even kidney involvement. Bladder disease can cause symptoms of dysuria, urinary urgency, hematuria, voiding dysfunction, or flank pain (49). However, DE of the ureter is usually silent without any specific urologic symptoms. If undetected, it may lead to fibrosis and compression of the ureter and ultimately hydronephrosis and loss of renal function, which makes ureteric disease very important to treat surgically (50). Removal of bladder endometriosis is primarily for symptom relief, while removal of ureteric disease is to prevent more significant clinical sequelae that can lead to loss of renal function. While no RCTs have evaluated if removal of urinary tract endometriosis improves fertility, observational data have shown favorable unassisted pregnancy rates (51, 52).

### Thoracic endometriosis

Thoracic endometriosis refers to the presence of endometriosis lesions in the lungs, pleura, or diaphragm and consists of four clinical presentations: catamenial pneumothorax, catamenial hemothorax, catamenial hemoptysis, and pulmonary nodules (53). While thoracic endometriosis has a high association with pelvic endometriosis and infertility, research on thoracic endometriosis and fertility is limited (54). Treatment is recommended due to the significant clinical sequalae of thoracic endometriosis and for symptom management, rather than for enhancement of fertility alone. The current consensus is for joint management between gynecologists and thoracic surgeons with surgical removal of lesions via video-assisted thoracic surgery and postoperative medical suppression (53).

### Surgical techniques for endometriosis and their impact on fertility

General principles in surgical management of endometriosis include lysis of preexisting adhesive disease, restoration of normal anatomy, and removal of all visible disease. In patients with endometriosis who desire future fertility, particular care is needed to minimize iatrogenic injury to the fallopian tubes and ovaries. Laparoscopy for removal of endometriosis should begin with thorough evaluation of the upper and lower abdomen and pelvis to identify anatomy and potential endometriosis lesions. After a thorough abdominal and pelvic survey has been performed, lysis of adhesions and removal of endometriosis can then be performed. Endometriosis lesions can be laparoscopically ablated, such as with electrocautery or carbon dioxide (CO2) laser, or excised with removal of affected peritoneum (2, 55). There is not a clear difference between the two approaches in terms of improvement of pain or pregnancy outcomes. However, excision is generally favored, especially for lesions in close proximity to critical structures such as the ureters and blood vessels to minimize risk of injury (30). The appearance and patency of the fallopian tubes should be assessed intraoperatively with chromopertubation.

For the removal of endometriomas, RCTs have shown a reduction in pain and recurrence rates postoperatively as well as increased rates of unassisted conception in patients undergoing cystectomy compared to ablation (40). The first step in cystectomy for endometrioma involves identifying the plane between the cyst wall and the normal ovary; careful identification is key to minimizing damage to healthy ovarian cortex. The incision is ideally made over the thinnest area over the endometrioma or at the antimesenteric border of the ovary. Multiple incisions to the ovarian cortex should be avoided (56). Dilute vasopressin may be used for hydrodissection to help with plane identification. Once the cyst wall is identified, traction and counter traction are utilized to carefully separate the cyst wall from the ovarian cortex. If bleeding is encountered, electrosurgery can be used sparingly (39). While effective at controlling bleeding, bipolar cautery can cause injury to adjacent healthy ovarian tissue and reduce ovarian reserve. Suture and hemostatic agents have been shown to reduce injury to ovarian reserve compared to electrosurgery. One RCT compared patients undergoing laparoscopy for unilateral endometrioma with bipolar cautery or suture and found that the suture group had significantly higher AMH levels at three months postoperatively (57). Similarly, another study showed a smaller postoperative reduction in AMH when hemostatic agents were used compared to bipolar cautery (58).

Due to the potential impact of adhesions on fertility from distorted pelvic anatomy, the use of anti-adhesion barriers has been explored in gynecologic surgery. A systematic review and meta-analysis of RCTs found that oxidized regenerated cellulose and collagen membrane with polyethylene glycol and glycerol were associated with a reduction in adhesion formation; however, none of these studies reported clinical outcomes such as pelvic pain or live birth rate (59). These studies were found to be of low quality. Overall, there has been no conclusive evidence on the use of anti-adhesions to reduce postoperative adhesions. However, since no adverse effects directly attributed to the use of anti-adhesion barriers have been identified, they could be used at the time of endometriosis surgery to reduce formation of postoperative adhesions (39).

### Regenerative therapies for endometriosis

Regenerative therapies for endometriosis are currently in experimental and preclinical stages. One of the more commonly investigated regenerative therapies are cell therapies, which involve administration of either autologous or allogenic living cell suspensions to repair or regenerate damaged tissues. Potential cell therapies for endometriosis are based on the principle of targeting various aspects of its pathogenesis. The inhibition of both focal angiogenesis and fibrosis, important components of endometriosis pathogenesis, could be therapeutic targets of cellular therapy (60). Mesenchymal stem cells have specifically been studied as a possible cell-based therapy for endometriosis because of their role in fibrosis, angiogenesis, and immune modulation. One study showed that adipose-derived stem cells suppressed stromal fibrosis and the growth of endometriosis-like lesions and inhibited the expression of pro-inflammatory and pro-fibrotic cytokines in a murine model of endometriosis (61). Other potential regenerative therapies include platelet rich plasma (PRP), which has been extensively studied in conditions such as Asherman’s syndrome and thin endometrium but more recently studied as a potential therapy for endometriosis (62, 63). Extracellular vesicles (EV), which are particles involved in intercellular communication, have also been identified as a possible therapeutic target for endometriosis (64). It is important to emphasize that all regenerative therapies for endometriosis are investigational, with almost all data coming from *in vitro* and preclinical animal studies. More research is needed to further develop these therapies for treatment of endometriosis, especially in the context of fertility enhancement.

## Discussion

In summary, surgical treatment of endometriosis for fertility enhancement is complex and depends on multiple clinical factors, including but not limited to the patient’s age, ovarian reserve, fertility goals (infertility history, ideal family size, desire/ability to do IVF), semen parameters, tubal status, and presence of any uterine pathology such as leiomyoma or adenomyosis (2, 39). The stage and location of disease will also guide treatment recommendations (**Table 1**). For superficial endometriosis associated with infertility, laparoscopic surgery for treatment of endometriosis may enhance fertility for unassisted pregnancy (2, 17, 28–30). However, current guidelines recommend against routine laparoscopic surgery prior to ART to improve live birth rates (17). Patients with deep endometriosis and infertility are likely to require ART over a trial of unassisted pregnancy, either as a primary treatment for infertility or immediately after surgery. Patients may be counseled towards surgery prior to ART if they have significant pain, presence of hydro/hematosalpinx, constriction of the ureter or bowel, or thoracic disease. Current guidelines do not recommend cystectomy for asymptomatic patients with endometriomas prior to IVF (2, 17). However, in some cases cystectomy may be needed first to facilitate oocyte retrieval.

**Table 1:** Key summary points for surgical management of endometriosis for fertility enhancement.

Key summary points
Superficial endometriosis
• For superficial endometriosis associated with infertility, laparoscopic surgery for treatment of endometriosis may enhance fertility for unassisted pregnancy
• Current guidelines recommend against routine laparoscopic surgery prior to ART to improve live birth rates until further studies are completed
• Laparoscopic surgery for treatment of endometriosis is a viable option for fertility seeking patients with superficial disease and symptoms related to endometriosis
Deep endometriosis
• There are no published RCTs that evaluate whether clinical pregnancy or live birth rates improve after surgical treatment of deep endometriosis
• Current guidelines do not recommend surgery before ART in patients with bowel endometriosis with a primary goal of improving fertility
• Patients with deep endometriosis and infertility are likely to require ART over a trial of unassisted pregnancy, either as a primary treatment for infertility or immediately after surgery
• Patients may be counseled towards surgery prior to ART if they have significant pain, presence of hydro/hematosalpinx, constriction of the ureter or bowel, or thoracic disease
Endometriomas
• Potential benefit of cystectomy for endometrioma include reduction in pain, facilitation of oocyte retrieval, prevention of possible endometrioma rupture, and detection of occult malignancy. Potential risks include diminished ovarian reserve
• In patients wishing to conceive with no other infertility factors are incidentally noted to have endometrioma without concern for malignancy, they can attempt unassisted conception for up to 6 months
• Current guidelines do not recommend cystectomy for asymptomatic patients with endometriomas prior to IVF
• If ovarian cystectomy is planned for endometrioma > 3–4 cm, oocyte/embryo freezing should be considered first if normal ovary is accessible for oocyte retrieval. In some cases, cystectomy may be needed prior to IVF to facilitate oocyte retrieval

Most treatment strategies for patients with endometriosis desiring fertility depend on the patient’s age, ovarian reserve, fertility goals (infertility history, ideal family size, desire/ability to do IVF), semen parameters, tubal status, and presence of any uterine pathology such as leiomyoma or adenomyosis. There is a lack of consensus on how to approach patients with endometriosis-associated infertility (EAI). The authors of this report approach EAI as following:

### Approach to EAI with superficial endometriosis

If there are no other infertility factors, then patients with superficial endometriosis can be treated as unexplained infertility. If patients are young (usually < 38 years old), then superovulation with clomid paired with intrauterine insemination (IUI) may be attempted for up to 3–4 cycles (2). If pregnancy is not achieved, the next step is IVF. As discussed previously, for infertility associated with superficial endometriosis, laparoscopy may enhance fertility for unassisted pregnancy, but current guidelines recommend against routine surgery prior to ART (2, 17, 28–30). However, if patients have significant symptoms related to endometriosis, then laparoscopy for treatment of endometriosis is a reasonable step prior to IVF.

### Approach to EAI with deep endometriosis and endometrioma

Treatment for infertility associated with deep endometriosis varies based on what organ is involved with disease, the presence of symptoms, patient age, ovarian reserve, and ability/desire to undergo ART. Typically, patients with deep disease also have significant pain and other symptoms due to endometriosis. For deep disease other than endometriomas, the decision to perform surgery with disease excision is beneficial for elimination of pain and could heighten natural fertility, provided the fallopian tubes are patent and there are no male factors contributing to infertility. After surgery, if at least one fallopian tube is patent, the patient is young with normal ovarian reserve, and no male factor is present, a trial of unassisted conception can be attempted for 3–6 months. If pregnancy is not achieved, fertility treatment should be recommended. The ideal treatment is IVF as a first step, but superovulation with clomiphene citrate paired with IUI may be offered if the patient wants to proceed with a stepwise fashion (provided the patient is < 38 years old with at least one patent fallopian tube). In patients 38 years old or older, tubal factor, and/or other infertility factors, IVF should be recommended as the first step. A potential exception to the recommendation for a surgery-first approach is in patients with endometriomas and diminished ovarian reserve, who may benefit from IVF and embryo banking first prior to surgical management due to the risk of ovarian damage and subsequent lack of response to IVF after surgery. There are no specific IVF protocols that have been shown to improve outcomes for patients with EAI undergoing IVF (17, 65, 66). GnRH agonists may be administered for 6–8 weeks prior to a frozen embryo transfer to suppress endometriosis lesions and reduce inflammation. This long suppression may not be needed in those who have had recent extensive surgical excision of disease and have no evidence of uterine adenomyosis.

## Conclusion

Endometriosis is a common, though incompletely understood, chronic disease that can impact fertility through multiple potential mechanisms. Imaging is key in diagnosing presence and extent of disease, though surgery with histopathology diagnosis is still considered diagnostic gold standard. While surgery with excision of disease followed by ovarian suppression is an adequate strategy for pain control, surgery for patients seeking to optimize fertility or for whom infertility is the only symptom is complex. While there may be potential improvements in fertility-related outcomes, this must be weighed against potential impacts to ovarian reserve. Surgical techniques that minimize damage to the ovaries, fallopian tubes, and uterus are essential when fertility is the goal. While there is increasing interest in regenerative therapies for endometriosis, these remain investigational and are not yet utilized for fertility enhancement in patients with endometriosis.
